# Efficient Identification of Monoclonal Antibodies Against Rift Valley Fever Virus Using High-Throughput Single Lymphocyte Transcriptomics of Immunized Mice

**DOI:** 10.3390/antib14010012

**Published:** 2025-02-04

**Authors:** Ronit Rosenfeld, Ron Alcalay, Yfat Yahalom-Ronen, Sharon Melamed, Avital Sarusi-Portuguez, Tal Noy-Porat, Ofir Israeli, Adi Beth-Din, Ronnie Blecher-Gonen, Theodor Chitlaru, Erez Bar-Haim, Tomer Israely, Anat Zvi, Efi Makdasi

**Affiliations:** 1 Department of Biochemistry and Molecular Genetics, Israel Institute for Biological Research, Ness-Ziona 74100, Israel; rona@iibr.gov.il (R.A.); taln@iibr.gov.il (T.N.-P.); ofiri@iibr.gov.il (O.I.); adib@iibr.gov.il (A.B.-D.); theodorc@iibr.gov.il (T.C.); erezb@iibr.gov.il (E.B.-H.); anatz@iibr.gov.il (A.Z.); efim@iibr.gov.il (E.M.); 2 Department of Infectious Diseases, Israel Institute for Biological Research, Ness-Ziona 74100, Israel; yfatyr@iibr.gov.il (Y.Y.-R.); sharonm@iibr.gov.il (S.M.); tomeri@iibr.gov.il (T.I.); 3 The Mantoux Bioinformatics Institute of the Nancy and Stephen Grand Israel National Center for Personalized Medicine, Weizmann Institute of Science, Rehovot 7632706, Israel; avital.sarusi-portuguez@weizmann.ac.il; 4 The Crown Genomics Institute of the Nancy and Stephen Grand Israel National Center for Personalized Medicine, Weizmann Institute of Science, Rehovot 7632706, Israel; ronnie.blecher@weizmann.ac.il

**Keywords:** antibody discovery, recombinant antibody, single-cell analysis, 10x Genomics, antibody repertoire profiling, Rift Valley fever virus (RVFV), RVFV Gn glycoprotein, RVFV Gc glycoprotein

## Abstract

**Background**: Rift Valley fever virus (RVFV) is a zoonotic virus that poses a significant threat to both livestock and human health and has caused outbreaks in endemic regions. In humans, most patients experience a febrile illness; however, in some patients, RVF disease may result in hemorrhagic fever, retinitis, or encephalitis. While several veterinary vaccines are being utilized in endemic countries, currently, there are no licensed RVF vaccines or therapeutics for human use. Neutralizing antibodies specifically targeting vulnerable pathogen epitopes are promising candidates for prophylactic and therapeutic interventions. In the case of RVFV, the surface glycoproteins Gc and Gn, which harbor neutralizing epitopes, represent the primary targets for vaccine and neutralizing antibody development. **Methods**: We report the implementation of advanced 10x Genomics technology, enabling high-throughput single-cell analysis for the identification of rare and potent antibodies against RVFV. Following the immunization of mice with live attenuated rMP-12-GFP virus and successive Gc/Gn boosts, memory B cell populations (both general and antigen-specific) were sorted from splenocytes by flow cytometry. Deep sequencing of the antibody repertoire at a single-cell resolution, together with bioinformatic analyses, was applied for BCR pair selection based on their abundance and specificity. **Results**: Twenty-three recombinant monoclonal antibodies (mAbs) were selected and expressed, and their antigen-binding capacities were characterized. About half of them demonstrated specific binding to their cognate antigen with relatively high binding affinities. **Conclusions**: These antibodies could be used for the future development of efficacious therapeutics, as well as for studying virus-neutralizing mechanisms. The current study, in which the single-cell sequencing approach was implemented for the development of antibodies targeting the RVFV surface proteins Gc and Gn, demonstrates the effective applicability of this technique for antibody discovery purposes.

## 1. Introduction

Rift Valley fever virus (RVFV), first identified in 1931 [1], is the etiological cause of a zoonotic disease that affects domestic and wildlife ruminants as well as humans in endemic regions, mainly in Africa. RVFV, a Phlebovirus belonging to the Phenuiviridae family, is categorized as an emerging pathogen. It is mainly transmitted by mosquitoes and has the potential to cause widespread outbreaks in diverse geographical regions due to the presence of its vector [2,3,4,5,6]. Its pandemic potential and the establishment of novel endemic regions are due to the globalization of the livestock trade and the presence of large and concentrated groups of virus-naive animal hosts. These concerns are further enhanced by the changing global weather patterns that may enable the spreading of RVFV-carrying mosquito populations to new geographic regions [7,8,9,10]. The transmission of RVFV from livestock to humans may occur through mosquito bites or through contact with contaminated tissues of infected livestock, often resulting in mild flu-like symptoms. However, severe progression may involve encephalitis, ocular disease, or potentially lethal hemorrhagic fever [11,12,13]. Additionally, RVFV infection during pregnancy may lead to a miscarriage through direct placental infection [14]. The World Health Organization (WHO) and the U.S. National Institutes of Health have acknowledged the high threat posed by RVFV to livestock and humans. To prioritize the research and development of vaccines and therapeutic countermeasures, the virus was included in the Blueprint list, together with other emerging viral pathogens such as Ebola virus, Zika virus, Lassa fever virus, Nipah virus, Crimean–Congo hemorrhagic fever virus, severe acute respiratory syndrome coronavirus, and Middle East respiratory syndrome coronavirus [15,16].

The RVFV genome consists of three segments: the small (S), medium (M), and large (L) segments [17]. The S segment encodes for the nucleoprotein (N) and the non-structural NSs protein, which acts as a major virulence factor, counteracting the innate immune response by blocking the activation of the IFN-β promoter [18,19]. The M segment encodes the structural glycoproteins Gn and Gc, as well as the non-structural proteins NSm and a 78 kDa protein. The Gn and Gc glycoproteins are located on the surface of the virion as heterodimers, which then assemble into higher-order structures, and are involved in viral attachment, entry, and fusion [20,21]. The L segment encodes the viral RNA-dependent RNA polymerase (RdRp), which synthesizes both viral mRNA and genomic RNA [19].

Several RVFV vaccines have been licensed for veterinary use, but these vaccines have suboptimal safety and efficacy [22,23,24,25] and none of them meet the requirements for human-use licensure. The conditionally licensed for use in livestock MP-12 live-attenuated vaccine strain was generated through the serial passage of the virulent ZH548 strain in the presence of the chemical mutagen 5-fluorouracil [26,27]. Next-generation rMP-12 candidate vaccines, including genetically modified strains lacking the NSs gene, are suggested to have a superior attenuation profile compared to other strain variants. Further optimization of the vaccination regimen to enhance immunogenicity is necessary for its application, both in humans and animals [28,29].

Naturally acquired neutralizing antibodies, primarily targeting Gc and Gn proteins, provide protection against RVFV in animals and humans [30,31,32]. Monoclonal antibodies (mAbs) represent prophylactic and therapeutic clinical strategiesto prevent infection and disease progression, as has been demonstrated for several viral diseases including human immunodeficiency virus [33], severe acute respiratory syndrome coronavirus 2 [34,35,36,37], and respiratory syncytial virus [38]. As for RVFV infections, potent pre-exposure protection and strong post-exposure therapeutic benefits were reported in animal models upon administration of mAbs that target the Gn and/or Gc proteins [39,40]. It should be noted that most of the studies reporting the isolation of neutralizing Abs against RVFV pointed out that of the two glycoproteins of the pathogen envelope, Gn is a primary target of the neutralizing-antibody response in both animals and humans [4,20,23,32,39,40,41,42].

Single-B cell isolation is a powerful technique for antibody discovery, which involves the direct isolation and cloning of antibody genes from individual B cells. This approach enables the conservation of the original pairing of the heavy and light chain variable regions and therefore promotes the preservation of the natural specificity and affinity of the isolated antibodies. In particular, the 10x Genomics platform together with high-throughput sequencing enables parallel analyses, providing extensive information on both antibody sequences and gene expression profiles of thousands of individual B cells [43,44,45]. The rapid isolation of antigen-specific binders may be facilitated when combining single-cell technology with antigen-specific cell sorting.

In the current paper, we report the successful implementation of this innovative 10x Genomics platform for antibody discovery, specifically toward the identification of antibodies directed against RVFV Gn and Gc surface glycoproteins which represent the main targets of the virus-neutralizing response.

## 2. Materials and Methods

### 2.1. RVFV Strain rMP-12

Recombinant RVFV strain rMP-12-GFP (referred to as rMP-12) was kindly provided by Prof. Tetsuro Ikegami from the University of Texas Medical Branch of Galveston (UTMB) [29]. The virus stocks were propagated and titered on Vero or Vero E6 cells. The handling of and working with rMP-12 were conducted in accordance with the biosafety guidelines of the Israel Institute for Biological Research (IIBR).

### 2.2. Recombinant Gc and Gn Protein (rGc and rGn) Production

The DNA sequence of RVFV strain MP-12 Segment M (GenBank: DQ380208.1) was used to design pcDNA3.1+-based expression plasmids mediating the recombinant expression of the MP-12 Gc glycoprotein ectodomain (Cys691-Ser1139) and MP-12 Gn glycoprotein ectodomain (Pro156-Thr581). The sequences for expression were codon-optimized and designed to contain an AviTag (to enable targeted mono-biotinylation by BirA), x6 His, and Strep tag II (to facilitate protein purification) at the product C-terminus.

The recombinant proteins (rGc and rGn) were expressed in CHO cells using the ExpiCHO^TM^ expression system (ThermoFisher Scientific, Waltham, MA, USA, A29127), followed by purification using HisTrap^TM^ (Cytiva, Marlborough, MA, USA, 17-5247-01). The purity of each antigen was evaluated by SDS-PAGE gel under non-reducing conditions. The purified proteins were stored in PBS at −20 °C.

Mono-biotinylated rGc (mono_bio-Gc) was prepared using a BirA biotin–protein ligase kit (GeneCopoeia, Rockville, MD, USA, BI001). Biotinylated rGc and rGn (bio-Gc and bio-Gn) were prepared using an EZ-Link^TM^ Sulfo-NHS-Biotin kit (ThermoFisher Scientific, Waltham, MA, USA, 21217). The excess free biotin was removed by buffer exchange using Amicon Ultra (Millipore, Cork, Irland, 501024).

### 2.3. Mice Immunization

Vaccination was performed through subcutaneous (s.c.) injections (0.1 mL/animal) of increasing doses of rMP-12 to female BALB/c mice (6–8 weeks) at approximately 1-month intervals. The prime vaccination was performed with 1 × 10^6^ pfu/animal, followed by 2 boosts of ~4 × 10^7^ pfu/mouse, and a third boost of 1 × 10^9^ pfu/mouse. Subsequently, the mice were subcutaneously boosted with recombinant RVFV glycoprotein [150 µg in 0.2 mL/animal; adjuvanted with Incomplete Freund’s adjuvant (IFA)]. Four mice (m1–m4) were boosted with rGc protein and one mouse (m5) was boosted three times, 4 weeks apart, with Gn protein. Serum samples were collected prior to the prime vaccination, and ~2 weeks following each vaccination. For B cell isolation, the mice were sacrificed, and spleens were removed for the preparation of single-cell suspensions. The animal vaccination was conducted in accordance with the guidelines of the IIBR Institutional Animal Care and Use Committee (M-18-23).

### 2.4. B Cell Isolation

Single-cell suspensions were prepared from immunized mice spleens using a standard protocol. Briefly, the spleens were carefully extracted, splenocytes were separated in PBS using a 70 µm cell strainer, and cell clumps were disrupted by pipetting to obtain a single-cell suspension. The splenocytes were washed by centrifugation at 300× *g* for 5 min and erythrocytes were lysed by hypotonic shock using Hybri-max^TM^ Red Blood Cell-Lysing Buffer (Sigma, St. Louis, MO, USA, R7757). Following an additional wash, the cells were resuspended in PBS/1% BSA and counted using a hemocytometer after staining with 0.1% Trypan blue.

**Figure 1:**
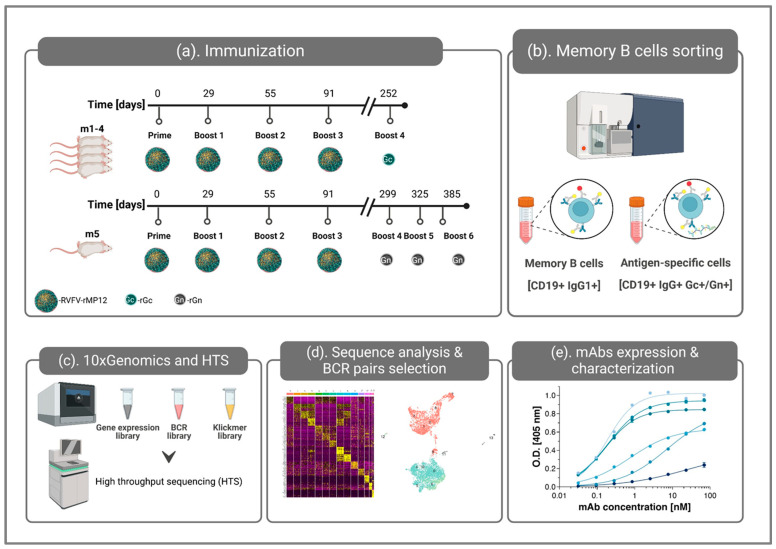
Single-cell BCR repertoire-profiling experimental approach implemented in the current study for identification of RVFV-specific antibody sequences. (**a**) The mouse immunization regimen consisted of priming and three boosts with live attenuated RVFV rMP-12, followed by recombinant antigen administration. (**b**) Eleven days after the last boost, splenocytes were isolated, stained, and subjected to sorting of IgG-presenting B cells and antigen-specific sub-populations. (**c**) Total transcriptome, full V(D)J, and capture barcode sequences (antigen-specific barcode acquired by the dCODE Klickmer^®^-PE reagent) were determined at the single-cell level using 10x Genomics technology and high-throughput sequencing (HTS). (**d**) The data analysis enabled the determination of phenotypic subsets of splenocytes and, most importantly, the selection of BCR pairs for recombinant expression. (**e**) The binding capacities of the expressed mAbs were characterized. The figure was created with BioRender.com.

Separate procedures were performed for Gc- and Gn-specific antibody discovery (see Figure 1).

### 2.5. Cell Staining and FACS 

B cells were resuspended in PBS/1% BSA at a concentration of 3–5 × 10^6^ cells/mL. Memory B cells were stained with anti-CD19-PE-Cy5 (eBioscience, San Diego, CA, USA, RRID AB_657672; 0.25 μL), anti-IgG1-APC, and anti-IgG2ab-APC (Miltenyi Biotec, Bergisch Gladbach, Germany, 130-095-838; 1 μL each). For enrichment of antigen-specific memory B cells, mono_bio-Gc coupled with dCODE^®^ Klickmer-PE (Immunodex, Copenhagen, Denmark, FBC0302,) or bio-Gn coupled with SA-PE (eBioscience, 12-4317-87) were added to the labeled cells. The cells were incubated for 30 min on ice, followed by washing and resuspension in 1 mL PBS/1% BSA for sorting in a BD FACSAria^TM^ III Cell Sorter (Becton Dickinson, Franklin Lakes, NJ, USA).

B cells were sorted according to the surface marker expression pattern CD19+/IgG+/Ag+, applying FSC-H/FSC-W-based duplet discrimination settings at a flow rate of 3.8–4.6 using an 85/100 μm nozzle. The sorted cells were collected into FACS tubes (Falcon, Corning, NY, USA) containing 1 mL DMEM/10% FCS. The cells were washed with 0.04% BSA (Invitrogen, Carlsbad, CA, USA, AM2616)/Ambion^TM^ nuclease-free water (Invitrogen, Austin, TX, USA, AM9937), counted (using a hemocytometer) and adjusted to a concentration of ~1000 cells/μL.

### 2.6. Library Preparation and Sequencing

Single-cell RNA sequencing (scRNA-seq) libraries were prepared at the Crown Genomics Institute of the Nancy and Stephen Grand Israel National Center for Personalized Medicine (G-INCPM), Weizmann Institute of Science, Israel, using 10x Genomics technology. Approximately 10,000 sorted cell suspensions per sample were processed with a Chromium Next GEM Single Cell 5′ v2 kit (10x Genomics, PN-1000263) according to the manufacturer’s protocol. Single-cell cDNA was separated into aliquots for the generation of three types of libraries: (i) a gene expression library, using a scRNA-seq library construction kit (10x Genomics, PN-1000190); (ii) a BCR profiling library (V(D)J-enriched library), using a Chromium Single-Cell Mouse BCR Amplification kit (10x Genomics, PN-1000255), and (iii) a dCODE^®^ Klickmer library (for Gc library only), using a Chromium 5′ Feature Barcode kit (10x Genomics, PN-1000541). The final libraries were quantified by qPCR using an NEB-next Library Quant kit (New England Biolabs, Ipswich, MA, USA, E7630) and iQuant dsDNA HS Assay Kit (ABP Biosciences, Beltsville, MD, USA, N011) The library fragment size was assessed using a Tapestation High-Sensitivity DNA Kit D1000 (Agilent, Santa Clara, CA, USA, 5067-5584). The libraries were sequenced on an Illumina NovaSeq6000, using an Sp 100 cycles kit (Illumina, San Diego, CA, USA, 20028401), allocating 800 M reads in total.

### 2.7. Data Analysis

#### 2.7.1. scRNA-seq Analysis

Cell Ranger (v7.1, 10x Genomics [46]) with default parameters was used for alignment (GRCm39-2024-A version, downloaded from 10x Genomics website), filtering, barcode counting, and UMI counting. The pipeline also performed sequence assembly and paired clonotype calling on the V(D)J libraries.

The Seurat package (v4.9.9; [47]) was used for the downstream analysis and visualization. Gene–cell matrices were filtered to keep cells that had less than 10% of their reads mapped to mitochondrial genes, between 250 and 6500 genes, and between 500 and 35,000 UMIs. In addition, genes detected in fewer than 10 cells were excluded from the analysis.

The expression data were normalized using Seurat’s NormalizeData function, which normalizes the feature expression measurements for each cell by the total expression, multiplies this by a scale factor (10,000), and then log-transforms the results. The top 2000 highly variable genes were identified using Seurat’s FindVariableFeatures function and the ‘vst’ method. Each cell was assigned a cell-cycle score using Seurat’s CellCycleScoring function and the NCBI’s orthologs of the G2/M and S phase marker lists from the Seurat package. Potential sources of unspecific variation in the data were removed by regressing out the mitochondrial gene proportion and UMI count using linear models and finally by scaling and centering the residuals, as implemented in the function “ScaleData” of the Seurat package. Principal component analysis (PCA) was performed. We selected 20 principal components (PCs) for the downstream analyses. Cell clusters were generated using Seurat’s unsupervised graph-based clustering functions “FindNeighbors” and “FindClusters” (resolution = 0.5). Uniform manifold approximation and projection (UMAP) was generated using “RunUMAP” on the projected principal component (PC) space. The Seurat functions “FeaturePlot”, “DimPlot”, “DotPlot”, and “VlnPlot” were used for visualization. The plots were further formatted using custom R scripts using the packages ggplot2 [48] and patchwork [49].

Marker genes for each cluster were identified by performing differential expression analysis between a distinct cell cluster and the cells of the other clusters using the non-parametric Wilcoxon rank sum test (Seurat’s FindAllMarkers function). SingleR [50] was used to annotate the cells using the ImmGenData as a reference. The clusters were annotated based on the SingleR results.

#### 2.7.2. BCR Sequence Analysis

The Cell Ranger BCR output was processed using the Immcantation toolbox (v4.5) and the docker container. The IgBLAST database was used to assign V(D)J gene annotations to the BCR FASTA files using the Change-O package [51], resulting in a matrix containing sequence alignment information for both the light and heavy chain sequences. The output was further filtered to remove nonproductive sequences, low quality cells, cells without associated constant region calls, or cells with multiple heavy chains. Clonotyping performed by applying the hierarchicalClones function in SCOPer [51]; sequences differing by a length normalized Hamming distance of 0.1 within the CDR3 region were defined as clones using single-linkage hierarchical clustering. This threshold was determined through manual inspection of distance to nearest neighbor plots using the SHazaM package [51]. These heavy chain-defined clonal clusters were further split if their constituent cells contained light chains that differed in their V and J genes. Within each clone, germline sequences were reconstructed using the createGermlines function in Dowser [52]. BCR mutation frequencies were then estimated using SHazaM. The BCR sequence data, clone assignments, and estimated mutation frequencies were integrated with the single-cell RNA-seq data by aligning and merging the data with the metadata slot in the processed RNA-seq Seurat object.

#### 2.7.3. dCODE^®^ Klickmer Analysis

The Klickmer library included a low number of reads and the 10x cellranger pipeline could not be applied to the data. Therefore, Klickmer sequences and cell barcodes were extracted from the fastq files. Similar to the regular 10x library, R1 contained the cell barcodes (16 first bps) and UMIs (10 last bps) and R2 contained the Klickmer sequence (FBC0302-GGTTCGACGCATACC). A total of 649 reads were present following filtering of R2 reads containing the FBC0302 sequence without mismatches. The cell barcodes and UMIs for these reads were also extracted from the R1 fastq files. Using the cell barcode, UMI sequence, and FBC0302 sequence data, a UMI count matrix was created for each cell barcode, resulting in a “Klickmer_count” value per cell. This information was further integrated with the BCR data for each cell (mainly the clone_id). Only cell barcodes that were also valid for the BCR analysis were included. Next, “Klickmer_clone_count” values, representing the total number of Klickmer molecules detected per clone, were calculated.

### 2.8. Recombinant mAb Production

Recombinant mAbs were produced on a small scale by Biointron Biotech Co., Ltd. (Shanghai, China; “RushMab” expression package). Selected pairs of antibody variable domains were gene-optimized, synthetized, and cloned into pcDNA3.4-based expression vectors encoding the constant domains of human IgG1/kappa. The recombinant mAbs were transiently expressed in CHO cells and had a purity of >95%, as assessed by reducing and non-reducing SDS-PAGE. Additionally, monomeric fractions of the expressed antibodies were measured using size-exclusion chromatography at 214 nm, which indicated the following monomeric IgG fractions: 92.8, 99.1, 96.5, 96.1, 99.6, 93.8, 97.3, 98.9, and 100% for Gc_1 to Gc_9 mAbs, respectively, and 100, 99, 97.5, 99.8, 99, 99.6, 99.2, 99.7, 99.1, 99, 99.5, 98., 96.1, and 88% for Gn_1–Gn_14 mAbs, respectively. Of note, similar results for the monomeric IgG fractions were measured for all tested antibodies at 280 nm.

### 2.9. Antigen-Binding Assays

Standard direct ELISAs were performed using microtiter plates coated with 2 μg/mL of recombinant protein (rGc or rGn) or 2 × 10^6^ PFU/mL of live RVFV rMP-12.

For mouse antibody detection, AP-conjugated donkey anti-mouse antibody (Jackson ImmunoResearch, West Grove, PA, USA, 175-055-150; used at 1:2000 dilution) was applied, followed by development with PNPP substrate (Sigma, St. Louis, MO, USA, N1891). Each serum sample binding assay was performed at least twice with technical duplicates.

For recombinant mAb detection, AP-conjugated donkey anti-human IgG antibody (Jackson ImmunoResearch, West Grove, PA, USA, 709-055-149; used at 1:4000 dilution) was applied, followed by development with PNPP substrate. The chimeric mAb HN_2, directed against BoNT/A toxin [53], was used as an isotype control.

Specificity ELISAs were performed (three technical repeats and at least twice per mAb) in serial dilutions. The plotted signals represent OD [405 nm] values against rGc, rGn, and BSA in the present of mAbs at a constant concentration [10 μg/mL]. To obtain comparative signals between tested plates, 100 ng/mL of mAb Gc_9 was used as a standard and binding signals were quantified when its signal reached 0.5 OD.

### 2.10. Biolayer Interferometry Assays

The binding kinetic parameters of the recombinant mAbs were determined using an Octet Red system (ForteBio, Fremont, CA, USA, Version 8.1, 2015) that measures biolayer interferometry (BLI). All steps were performed at 30 °C with shaking at 1500 rpm in a black 96-well plate containing 220 μL of solution in each well. Streptavidin-coated biosensors (Sartorius, Göttingen, Germany, 18-5019) were loaded with biotinylated antigen (3 μg/mL) to reach a 2.5–4 nm wavelength shift, followed by a wash. The sensors were then reacted for 300 s with each mAb at serial concentrations ranging from 100 nM to 1.56 nM (association phase) and then transferred to buffer-containing wells for another 300 s (dissociation phase). Binding and dissociation were measured as the changes in light interference over time after subtraction of parallel measurements from unloaded bio-sensors. Sensorgrams were fitted with a 1:1 binding model using the Octet data analysis software 8.1. The presented values are an average of at least two repeated measurements.

## 3. Results

### 3.1. Study Design and Mice Immunization

To isolate RVFV-specific antibodies, advanced 10x Genomics technology, enabling gene expression and immune profiling at the single-cell level, was applied. The experimental design of the study is schematically depicted in Figure 1. The vaccination regimen (see panel a), in order to maximize the subsequent yield of relevant antibodies, consisted of administration of the live attenuated RVFV rMP-12, according to an immune regimen of priming, followed by three boosts 4 weeks apart. In addition, consistent with the fact that the viral surface glycoproteins Gc and Gn are the main proteins mediating neutralization [4,20,23,32,39,40,41,42], the mice were further boosted with recombinant versions of Gc or Gn (rGc and rGn). Thus, four mice (referred to as m1–m4) were boosted with rGc once, while another mouse (m5) was administered with rGn 3 times, due to an observed lower immune response.

**Figure 2:**
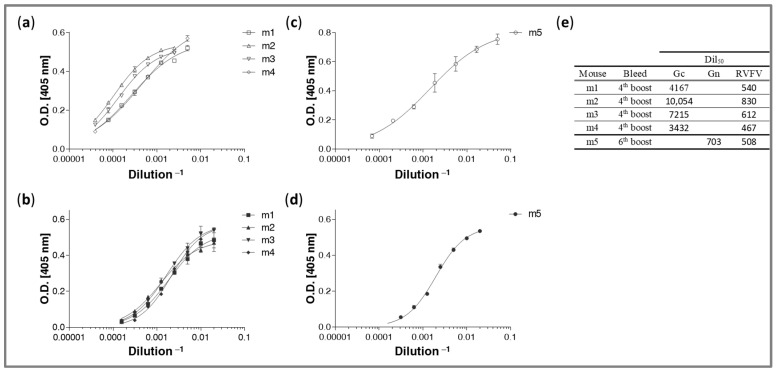
Specific humoral responses of RVFV-immunized mice. ELISA assessments of polyclonal reactivity of sera collected from mice prior to spleen harvesting (11 days after last boost). (**a**,**b**) The binding capacities against rGc (**a**) and whole live virus (**b**) of the serum samples collected from m1–m4. (**c**,**d**) The binding capacities against rGn (**c**) and whole live virus (**d**) of the serum samples collected from m5. The data represent average of duplicates ±SD. (**e**) The calculated Dil50 values (the dilution factor at 50% of maximal binding) of each tested serum sample.

The antibody response elicited in vaccinated animals against live RVFV rMP-12, rGc, and rGn was evaluated by ELISA throughout the immunization period, as well as after the last boost, prior to collection of spleens. As presented in Figure 2, strong rGc binding (panel a) was demonstrated by the serum samples collected from mice m1–m4 (boosted with rGc). They also demonstrated binding to live whole virus (panel b) to a lesser extent (approx. one order of magnitude); the Dil50 values (the dilution factor at 50% of maximal binding) are indicated in the table in panel e. The binding levels of mouse m5’s (boosted with rGn) serum to rGn and live whole virus were similar (Figure 2c–e). Of note, throughout the immunization schedule, the response elicited against rGc was consistently higher than that against rGn. This observation may indicate that Gn exhibits inferior immunogenicity compared to the Gc protein. Although ExpiCHO cells generally produce human-like sialylated proteins, some heterogeneity pertaining to their glycosylation status may exist. Accordingly, it is possible that the observed variation in the immune response induced by the two antigens is due, at least in part, to changes in the glycosylation pattern of the recombinant proteins. This aspect was not further interrogated in the current study.

### 3.2. Sample Preparation for Single-Cell Sequencing

Eleven days after the last boost, the immunized mice’s spleens were harvested, and splenocytes were isolated. For the enrichment of specific cell populations by fluorescence-activated cell sorting (FACS), the cells were labeled with antibodies for CD19 and IgG1/2 for the identification of memory B cells. To enable the sorting of antigen-specific populations, the cells were also stained with labeled antigens. In the case of the Gc-boosted mice (to be referred to as “Gc-sample”), dCODE Klickmer^®^ technology was implemented. This technology allows for the association of deep genomic profiles with antigen recognition at the single-cell level based on capture barcodes [54]. Accordingly, the Gc-sample cells were stained with mono-biotinylated rGc (mono-bio-Gc) attached to the dCODE Klickmer^®^-PE reagent. For Gn-specific B cell identification, the cells of the Gn-boosted mouse (to be referred to as “Gn-sample”) were labeled with biotinylated rGn (bio-Gn) attached to SA-PE (see Figure 1b for a schematic description of the protocol).

For further processing, the two sorted populations of CD19+ and IgG+ cells (memory B cells) and cells that were also stained with a labeled antigen (antigen-specific memory B cells) were combined. The post-sorting analyses confirmed the effective enrichment of the desired cell populations consisting mostly (>90%) of memory B cells, 25% of which represented antigen-specific binding cells.

### 3.3. Single-Cell Gene Expression Profiling

Approximately 10,000 cells per sample were processed using 10x Genomics Chromium™ technology for the subsequent preparation of libraries and high-throughput sequencing. The scRNA-seq resulted in gene expression profiles for 6113 Gc-sample cells and 4672 Gn-sample cells. The quality control metrics indicated that the libraries were high-quality with a median Q30 score of >95% and a low percentage of reads mapping to mitochondrial genes, indicating minimal cell stress or damage.

**Figure 3:**
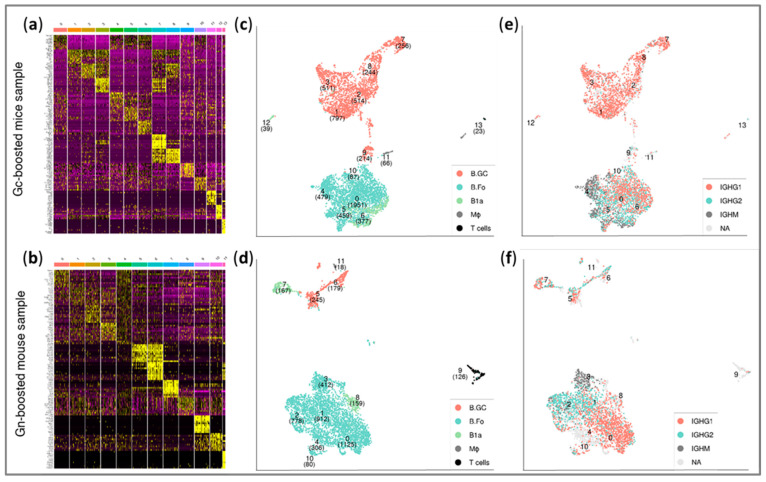
Single-cell phenotypic profiling. Gene expression profiling (scRNA-seq) using 10x Genomics platform and unsupervised cell clustering was performed according to differentially expressed markers (using Seurat). (**a**,**b**) Heatmaps of the top 10 differentially expressed genes in each cluster of the Gc-sample and Gn-sample, respectively. (**c**,**d**) UMAP visualization of individual cells (each point represents a single barcode, which corresponds to a single cell) in the Gc-sample and Gn-sample, respectively. The plots indicate the cluster numbers as well as the number of cells in each cluster (in brackets). Each cell is colored according to its annotation: B.GC, germinal center B cell; B.Fo, follicular B cells; B1a cells; MΦ, macrophages; and T cells. (**e**,**f**) UMAPs indicating cells expressing immunoglobulin heavy chain isotypes (IGHG1, IGHG2, and IGHM) in the Gc-sample and Gn-sample, respectively. NA indicates cells with no BCR information or isotypes represented by less than 1% of the cells.

The analyzed cells were clustered based on their differential expression profile, as indicated by the heatmaps (for Gc-sample and Gn-sample, Figure 3a and Figure 3b, respectively) showing the top 10 differentially expressed genes per cluster. Two-dimensional visualization of the scRNA-seq-based cell clusters was performed using uniform manifold approximation and projection. The UMAP plots for the Gc-sample and Gn-sample (Figure 3c and Figure 3d, respectively) have the scRNA-seq profiles, as well as the cell-type annotations, embedded. Two prevailing groups of close clusters could be distinguished, each composed mainly of either germinal center B cells (B.GC) or follicular B cells (B.Fo). This distinction of two cluster groups was observed in both the Gc-sample (Figure 3c; upper clusters: 1–3, 7, and 8; lower clusters: 0, 4–6, and 10) and the Gn-sample (Figure 3d; upper clusters: 5–6; lower clusters: 0–4, 8, and 10). Additional annotated cell types, B1a, macrophages (MΦ), and T cells, were present at substantially lower proportions, comprising less than 8% of the analyzed cells. It is noteworthy that while the Gc-sample was composed of comparable fractions of the main two cell types (about 53% B.Fo and 40% B.GC, Figure 3c), the Gn-sample was mainly composed of B.Fo cells (about 83%, Figure 3d).

### 3.4. Single-Cell BCR Profiling

In addition to gene expression profiling, high-throughput full V(D)J sequencing and analysis were performed for the two single-cell samples. B cells in which productive BCRs were not detected, cells without detected a constant region, as well as cells exhibiting multiple heavy chains were filtered out. Following this curation process, paired heavy and light chain sequences were successfully recovered from 5114 Gc-sample cells and 3310 Gn-sample cells (representing about 84% and 71% of the sequenced cells, respectively). Cell differences in the expression of immunoglobulin heavy chain isotypes were embedded into the UMAPs depicted in Figure 3e (Gc-sample) and Figure 3f (Gn-sample). About 85% of the sequenced cells expressed IgG1 or IgG2, demonstrating the efficient sorting of the cells using anti-IgG1/2-APC labeling.

The BCR pair clonotyping analysis performed based on the VH sequence similarity resulted in a total of 550 and 299 distinct clones with two or more cells per clone for the Gc-sample and the Gn-sample, respectively.

Following immunization, antigen-activated B cells undergo a process of clonal selection and expansion from pre-existing naive and memory B cells [55,56]. Consequently, the enriched B cell populations are expected to generate high-affinity neutralizing antibodies. Accordingly, the enrichment of B cells was calculated based on the number of cells in each particular clone (to be referred to as “clone_count”).

### 3.5. BCR Pair Analysis and Selection for Expression from the Gc-Sample

To identify the most commonly expressed BCR pairs in the Gc-sample, the analysis data were first sorted according to clone_count. The six BCR pair clones with the highest values for clone_count, with 68, 31, 28, 26, 31, and 35 cells per clone, respectively, were selected for subsequent expression as recombinant mAbs (referred to as Gc_1 to Gc_6; Table 1). An additional sorting step was performed according to “Klickmer_clone_count” (defined as the number of Klickmer molecules detected per clone) to facilitate the identification of clones that potentially specifically recognize the RVFV Gc protein. This latter sorting step resulted in the selection of six BCR pairs, representing clones with the highest values for Klickmer_clone_count and a clone_count higher than 10 (referred to as mAbs Gc_9, Gc_6, Gc_8, Gc_4, Gc_1, and Gc_7; Table 1), with total Klickmer counts per clone of 26, 18, 14, 10, 9, and 7, respectively. Notably, mAbs Gc_1, Gc_4, and Gc_6 were independently selected by both sorting procedures. Therefore, a total of nine BCR pairs, detailed in Table 1, were selected for expression as recombinant mAbs. The table details each mAb’s name, clone_id origin, clone_count, Klickmer_count, cluster origin, cell annotation, Ab isotype, and average number of mutations in the VH region compared to the original germline.

### 3.6. BCR Pair Analysis and Selection for Expression from the Gn-Sample

The Gn-sample BCR pairs were analyzed essentially as described above for the Gc-sample. However, although both samples were sorted to enrich antigen-specific memory B cells, the Gn-sample did not include a genetic barcode for the identification of cells that were labeled with the specific antigen. Accordingly, the BCR pairs selection was exclusively based on their abundance. As detailed in Table 2, fourteen BCR pair clones were selected, which had the highest values for clone_count, between 20 to 90 cells per clone (referred to as mAbs Gn_1-14, Table 2). In contrast to the selected BCR pairs from the Gc-sample, most of the selected clones from the Gn-sample were annotated as B.Fo. This observation is in agreement with the two samples’ phenotypic profiles, which showed a much lower fraction of B.GC-annotated cells within the Gn-sample compared to the Gc-sample.

### 3.7. Expression and Functional Validation of Recombinant mAbs

The sequence information of the germline gene origin and CDR3 amino acid sequence of the selected mAbs are provided in Table 3 for the heavy and the light chains. The data show that among the Gc_mAbs, Gc_4, Gc_6, and Gc_8 share a common germline origin for both the VH and VΚ segments (IGHV4-1 and IGKV6-17), while mAbs Gc_5 and Gc_9 share a common germline origin only for the VH segment (IGHV5-9). In the case of the Gn_mAbs, Gn_1, Gn_5, and Gn_10 share a common germline origin for the VH segment (IGHV9-3) and a close VK origin (IGKV3-5 or IGKV3-4). Overall, 23 chimeric recombinant mAbs, listed in Table 3, were expressed and purified based on the selected variable domain sequences and human IgG1/k constant domains.

**Figure 4:**
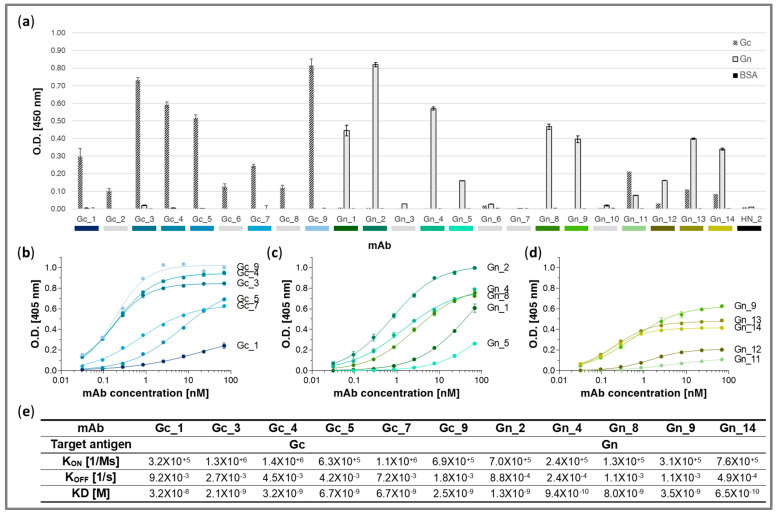
Characterization of Gc-sample and Gn-sample recombinant mAbs. (**a**) The specificity of the recombinant mAbs selected from the Gc-sample (Gc_mAbs) or Gn-sample (Gn_mAbs) was assessed by ELISA against rGc, rGn, and BSA. The chimeric antibody HN_2 was used as an isotype control. The data represent an average of technical triplicates ±SD and are representative of at least two independent experiments. (**b**,**d**) The reactivity profiles of the indicated mAbs, as determined by ELISA, against Gc ((**b**) Gc_mAbs) or Gn ((**c**,**d**) Gn_mAbs). The values represent the average of technical triplicates ±SD. (**e**). The kinetic binding parameters of the indicated mAbs were determined using biolayer interferometry. Increasing concentrations of each mAb were incubated with immobilized biotinylated rGc or rGn. The binding sensograms were fitted using the 1:1 binding model.

The recombinant mAbs were assessed for their binding capabilities (Figure 4). Specificity ELISAs were performed against rGc, rGn, and BSA (Figure 4a). Of the nine tested Gc_mAbs, six exhibited significant specific binding to rGc in the following order of binding capacity: Gc_9 > Gc_3 > Gc_4 > Gc_5 > Gc_1 > Gc_7; there was no detected binding to rGn or BSA. Low binding signals were observed for Gc_6, Gc_8, and Gc_2. As for the Gn_mAbs, significant rGn binding was detected for 10 mAbs in the following order of binding capacity: Gn_2 > Gn_4 > Gn_8 ~ Gn_1 > Gn_9 ~ Gn_13 > Gn_14 >> Gn_5 > Gn_12 > Gn_11. While no BSA binding signal was detected, Gc binding to some extent was observed for Gn_11, Gn_13, and Gn_14. Notably, Gn_11 exhibited stronger binding to rGc than rGn. No significant binding by a non-relevant chimeric mAb (anti-BoNT/A toxin HN_2 [53]), which was used as an isotype control, was observed.

To further characterize the mAbs’ binding capacity, the reactivity profiles of individual mAbs were assessed by half-maximal binding ELISA (Figure 4b–d), which can indicate their maximum binding capacity (B max) and apparent KD value (corresponding to the mAb concentration at half B max). Antibodies Gc_3, Gc_4, and Gc_9 demonstrated the highest binding to rGc with comparable calculated apparent KD values. Gc_7 and Gc_5 evidenced relatively moderate affinities, and Gc_1 exhibited the lowest affinity (Figure 4b). The binding curves of the Gn_mAbs, which also indicate the B max and apparent affinity values, with Gn_14, Gn_13, Gn_9, and Gn_2 showing the highest affinities, followed by Gn_12 ~ Gn_4, Gn_8, and Gn_11, and Gn_1 and Gn_5 presenting the lowest binding capacities (Figure 4c,d).

Next, kinetic antigen binding parameters were determined for the six Gc_mAbs and five Gn_mAbs with the highest Bmax and apparent KD values (see above) using biolayer interferometry (BLI). The K_on_, K_off_, and KD values calculated for each antibody are provided in Figure 4e. The measured binding kinetic parameters indicated a high affinity for all the tested mAbs. The affinities of the Gc_mAbs ranged from 2.1 × 10^−9^ M to 3.2 × 10^−8^ M, with mAb Gc_3, Gc_9, and Gc_4 showing the highest affinities and mAb Gc_1 showing the lowest. The KD values of the Gn_mAbs ranged from 6.5 × 10^−10^ M to 8 × 10^−9^ M, with mAb Gn_14 and Gn_4 exhibiting sub-M affinities and Gn_8 expressed the lowest affinity. Therefore, the affinities of the mAbs were in line, in most of the cases, to their previously determined binding profiles, particularly in the case of the Gc_mAbs.

## 4. Discussion

Despite the threat posed to public health by RVFV including its pandemic potential, either naturally or due to malicious use [9,57,58], there are no RVFV-specific vaccines or therapies that are approved for human use [28]. Monoclonal antibodies offer a promising approach for combating this potentially severe zoonotic disease. Single-B cell screening has recently served as an approach of choice in the analysis of the immune response elicited by various infections, as well as for isolating antibodies for therapeutic purposes [59,60,61,62].

With the objective of anti-RVFV antibody discovery, we present in this report the application of the highly accessible scRNA-seq 10x Genomics platform combined with advanced bioinformatic tools for the simultaneous processing and analysis of expression and BCR profiling at the single-cell level. Indeed, the approach detailed here successfully yielded several mAbs that exhibit high antigen binding abilities, which could serve as the basis for future studies addressing the development of efficacious therapeutic countermeasures. In brief, mice were immunized with the live RVFV rMP-12 strain and boosted with recombinant proteins (rGc or rGn) prior to the cell sorting and scRNA-seq analysis (see Figure 1a). The immunization schedule was designed to promote antigen-specific B cell activation and expansion of relevant B cell clones [44,63,64,65]. The subsequent FACS (of memory B cells in general and specific B cell in particular) was applied for the enrichment of antigen-specific cell populations which were used for the identification of highly specific mAbs (Figure 1b). Following phenotypic expression and BCR profiling (Figure 1c), highly prevalent sequences were selected as putative antigen-specific BCRs (Figure 1d), which were then expressed as recombinant mAbs to corroborate their antigen specificity (Figure 1e). As could be expected, due to the rGc/Gn boosts prior to the B cell isolation, the majority of the recombinant mAbs were found to be directed toward these surface glycoproteins. As for the Gc-sample, BCR pairs were also selected according to the antigen-specific barcode (dCODE^®^ Klickmer). Although no direct correlation was observed for binding capacity with the clone/Klickmer_counts, the mAb selection according to each of the parameters allowed for the identification of quality binders (Figure 4).

Profiling of the various B cell populations (from the analysis of individual cell transcriptomes) indicated different prevalences of cell types in the Gc-sample and Gn-sample. Interestingly, while the Gc-sample was composed of comparable fractions of the two main cell types (about 53% B.Fo and 40% B.GC), the Gn-sample was mainly composed of B.Fo cells (about 83%). Accordingly, most of the Gc-sample BCR pairs selected for subsequent expression were derived from cells annotated as B.GC (clusters 1, 2, 3, 7, and 8; see Figure 3 and Table 1) and were the IgG1 isotype. However, one clone (clone 2486, which served as the basis for mAb Gc_9), that was selected based on its Klickmer clone_count was composed of B.Fo cells (clusters 0, 5–6, and 10; Figure 3 and Table 1) and was the IgG2 isotype. In the case of the Gn-sample, most of the selected BCR pairs were the IgG1 isotype and belonged to B.Fo cells (cluster 0; Figure 3 and Table 2) except for two IgG2 clones (Gn_7 and Gn_10), one B.GC-derived clone (Gn_7), and two clones composed of both B.GC and B.Fo cells (Gn_1 and Gn_9). The subsequent binding characterization of the expressed mAbs (see Figure 4) indicated that no advantages in terms of binding capacity could be attributed to the cell type origin or antibody isotype. Taken together, our data suggest that binding characteristics cannot be predicted based on clone profiling, and therefore, it is not recommended to rely solely on cell annotation or antibody isotype information to select mAbs, and that binding abilities should be assessed empirically. Future studies of larger B cell repertoires may provide a better basis for the prediction and rational selection of specific mAbs. Conversely, the study found that the prevalence of clones exhibiting a particular BCR sequence represents a valuable parameter for selecting the relevant antibody.

Regarding the use of single-B cell screening for antibody discovery, it is important to point out that this approach has substantial advantages over classical methods (such as hybridomas, phage display, and REP-seq technologies) in term s of efficacy, time, and effort, and notably preserving Ab chain pairing [44,59], contributing to the quality of the resulting immunoglobulin products. Despite the prominent advantages of this method, one should note that the number of analyzed cells is often limited. Furthermore, this method may preferentially promote the selection of immunodominant epitopes rather than those involved in the pathogenesis of the virus. These limitations could be reduced by designing the immunization process to stimulate the response against antigens expressed in the course of infection, representing bona-fide virulence factors, and preselecting the appropriate B cell subset for the subsequent selection of mAbs (as recently reported in studies aimed at isolating human mAbs against Zika, RSV, and SARS-CoV-2 [61,62,66]). Performing these steps prior to the selection may optimize both the diversity and binding ability of the selected mAbs.

To conclude, of the 23 mAbs selected in the present study, 6 anti-Gc and 5 anti-Gn mAbs exhibited high binding abilities and will be further investigated for their virus-neutralizing potential in future studies. Moreover, full humanization of the mouse monoclonal antibodies as well as further affinity maturation may be required for potential therapeutic use. Our work underscores the applicability of 10x Genomics technology in accelerating antibody discovery efforts for combating emerging viral threats.

## Figures and Tables

**Table 1:** Selected Gc_BCR pair clones for expression as recombinant mAbs.

mAb	Clone_id	Clone Count	Klickmer Clone_Count	Clusters	Cell Annotation *	Isotype *	Mutation Count [Mean]
Gc_1	862	68	9	1, 2, 3, 7, 8	B.GC	IgG1/k	20.4
Gc_2	621	31	3	1, 2, 3, 7, 8	B.GC	IgG1/k	4
Gc_3	1685	28	5	1, 2, 3, 7, 8	B.GC	IgG1/k	3.6
Gc_4	1611	26	10	1, 2, 3, 7, 8	B.GC	IgG1/k	1.8
Gc_5	2314	31	3	1, 3, 7, 8, 9	B.GC	IgG1/k	1.8
Gc_6	1632	35	18	1, 2, 3, 7, 8	B.GC	IgG1/k	1.3
Gc_7	233	16	7	1, 2, 3, 7, 8	B.GC	IgG1/k	13.2
Gc_8	1633	11	14	1, 2, 3, 7	B.GC	IgG1/k	1.3
Gc_9	2486	18	26	0, 5, 6, 10	B.Fo	IgG2/k	12.1

* Predominant cell annotation and antibody isotype of the cells within one clone.

**Table 2:** Selected Gn_BCR pair clones for expression as recombinant mAbs.

mAb	Clone_id	Clone Count	Clusters	Cell Annotation *	Isotype *	Mutation Count [Mean]
Gn_1	695	90	0, 5, 6	B.GC/B.Fo	IgG1/k	4.5
Gn_2	1501	68	0	B.Fo	IgG1/k	6.2
Gn_3	1164	67	0	B.Fo	IgG1/k	27.8
Gn_4	247	57	0	B.Fo	IgG1/k	10.4
Gn_5	677	41	0	B.Fo	IgG1/k	6.1
Gn_6	535	30	0	B.Fo	IgG1/k	16.9
Gn_7	1105	30	5, 6, 7	B.GC	IgG2/k	5.8
Gn_8	969	27	0, 8	B.Fo	IgG1/k	20.5
Gn_9	1554	25	0, 5, 6	B.GC/B.Fo	IgG1/k	6.8
Gn_10	1613	25	0, 1, 2	B.Fo	IgG2/k	5.8
Gn_11	1457	24	0, 1, 3, 6, 7	B.Fo	IgG1/k	19.6
Gn_12	1859	22	0	B.Fo	IgG1/k	7.9
Gn_13	1131	21	0	B.Fo	IgG1/k	20.9
Gn_14	604	20	0	B.Fo	IgG1/k	9.4

* Predominant cell annotation and antibody isotype of the cells within one clone.

**Table 3:** Sequences of selected BCR pair for expression as recombinant mAbs.

mAb	VH Gene	HCDR3	VL Gene	LCDR3
Gc_1	IGHV1-18	ARGDYRGGTMDY	IGKV3-7	QHSWEIPHT
Gc_2	IGHV1S135	AREGGFAY	IGKV4-63	FQGSGYPLT
Gc_3	IGHV5-12-2	ARHGSSYWYFDV	IGKV4-72	QQWSSNPPT
Gc_4	IGHV4-1	ARRGNYVFAY	IGKV6-17	QQHYSTPFT
Gc_5	IGHV5-9	ARPYGNYLYYFDY	IGKV13-85	QQYWTTPYT
Gc_6	IGHV4-1	ARRRNYAMDY	IGKV6-17	QQHYSSPRT
Gc_7	IGHV1-80	ARRRNFAMDY	IGKV6-17	QQHYSTPLT
Gc_8	IGHV4-1	ARRGNYVMDY	IGKV6-17	QQHYSTPRT
Gc_9	IGHV5-9	GRWGGNYAWFDY	IGKV12-41	QHFWSTPWT
Gn_1	IGHV9-3	MTTVVADAMDY	IGKV3-5	QQSNEDPYT
Gn_2	IGHV2-6-7	DVGGYGVHWYFDV	IGKV1-117	FQGSHVPWT
Gn_3	IGHV1-18	SGSYDGFPYFDY	IGKV3-7	QHSWEIPYT
Gn_4	IGHV1S135	EDGNYGDY	IGKV12-46	QHFWGTPRT
Gn_5	IGHV9-3	LTTVADYFDY	IGKV3-5	QQSNEDPFT
Gn_6	IGHV8-8	IDPPEEAMDY	IGKV4-59	QQWSSSSLT
Gn_7	IGHV1S34	EDYRYEGYGMDY	IGKV6-15	QQYNSYPYT
Gn_8	IGHV1-85	FDYYGPWFAY	IGKV6-32	QQYYSSPWT
Gn_9	IGHV2-9-2	AWLPPYYAMDY	IGKV3-2	QQSKEFPWT
Gn_10	IGHV2-9	GGIPYAMDY	IGKV10-96	QQGNTLPYT
Gn_11	IGHV2-5	KDGSWFAY	IGKV4-74	HQYHLSPPT
Gn_12	IGHV5-17	RSYYGYVGY	IGKV6-23	HQYHLSPPT
Gn_13	IGHV1-22	DYAGAFDY	IGKV3-7	HHSWEIPRT
Gn_14	IGHV9-1	YGDYPFAY	IGKV6-15	QQYNSYPLT

The heavy and light variable region germline origins (VH and VL genes) and their respective CDR3 amino acid sequences for the 23 antibodies selected for recombinant expression.

## Data Availability

HTS data, generated in this study, have not been deposited in a public repository due to ongoing analysis. However, the raw data files may be available from the corresponding author upon reasonable request, which will be reviewed by the author and will be subjected to approval by the institute ethics committee to ensure compliance with privacy and data protection regulations.
